# Phylogenetic analysis and characterization of the complete chloroplast genome of *Dipsacus asperoides*, the endemic medicinal herb in China

**DOI:** 10.1080/23802359.2019.1640648

**Published:** 2019-07-18

**Authors:** Tianyi Cao, Jun Fei, Gang Zu, Guihe Han, Zhen Lai, Ning Ren, Quan Zhang

**Affiliations:** a Second Clinical Medical College, Zhejiang Chinese Medical University, Hangzhou, Zhejiang, China;; b Department of Orthopaedics, Zhejiang Integrated Traditional Chinese and Western Medicine Hospital, Hangzhou, Zhejiang, China;; c Hangzhou Xiaoshan Ruan Health Management Co., Ltd, Hangzhou, Zhejiang, China

**Keywords:** *Dipsacus asperoides*, Dipsacaceae, medicinal herb, chloroplast genome, phylogenetic analysis

## Abstract

*Dipsacus asperoides* is a traditional and endemic medicinal herb in China. Its roots was considered top grade herb as early as in the Shen Nong Herbal Classic. In this study, we assembled and analyzed the complete chloroplast genome of *D. asperoides*. The complete chloroplast genome is 160,481 bp in length, exhibiting a large single-copy region (88,546 bp), a small single-copy region (19,671 bp), and two inverted-repeat regions (26,132 bp in each one). The chloroplast genome of *D. asperoides* contains 133 genes, including 89 protein-coding genes (PCGs), 36 transfer RNA (tRNAs), and eight ribosome RNA (rRNAs). The overall nucleotide content of the chloroplast genome is A of 30.2%, T of 31.0%, C of 19.7%, and G of 19.1%, with a total AT content of 61.2% and GC content of 38.8%. However, the phylogenetic Maximum-Likelihood (ML) analysis based on the amino acid sequences of 89 PCGs from 14 species chloroplast genome that *D. asperoides* is closely related to *Dipsacus asper.* This study can be used for medicinal herb value research and clinical drug development.

*Dipsacus asperoides* has been used in China as an important traditional Chinese medicinal plant and it is belongs to the genus Dipsacus family Dipsacaceae. Its root named ‘Dipsaci radix’ from the capacity to heal broken bone. The genus *Dipsacus* includes about 15 species of tall herbaceous biennial plants growing to 1–2.5 m tall. Dipsacus species are native to Europe, Asia, and northern Africa (Zhang et al. 1997). *Dipsacus asperoides* is distributed widely in the southwestern of China and has been used as the medicinal herb to treat lumbar and knee pain, liver dysfunction, traumatic hematoma, threatened abortion, and bone fractures (Cong et al. 2013; Huang et al. 2014). However, there has been no study on the genomic data of *D. asperoides* that limited to research genetic engineering and natural populations of this species. We presented the complete chloroplast genome of *D. asperoides* and studied the phylogenetic relationship, which can use for medicinal herb value research and clinical drug development in further.

The specimen sample of *D. asperoides* was collected from Second Clinical Medical College of Zhejiang University of Traditional Chinese Medicine in Hangzhou, Zhejiang, China (30.28N, 120.15E). Total chloroplast (cp) DNA of *D. asperoides* was extracted from the tissue of roots using the modified CTAB method and stored in Second Clinical Medical College of Zhejiang University of Traditional Chinese Medicine (No. SCMC-ZJU-TCM-01). The whole cpDNA was purified and fragmented using the TAKARA Next Ultra™ II DNA Library Prep Kit (TAKARA, DL, and CN), that the cpDNA was sequenced. Quality control was performed to remove low-quality reads and adapters using the FastQC Version 0.11.8 (Andrews 2015). The chloroplast genome was assembled and annotated using the MitoZ (Meng et al. 2019). The physical map of the chloroplast genome was drawn using OrganellarGenomeDRAW Version 1.3.1 (Greiner et al. 2019). The complete chloroplast genome of *D. asperoides* was annotated and submitted to NCBI of the GenBank that accession number is MK809975.

The complete chloroplast genome of *D. asperoides* is the circular and the length of 160,481 base pairs (bp), which exhibiting a characteristic quadripartite structure with a large single-copy region (LSC) of 88,546 bp, a small single-copy region (SSC) of 19,671 bp, and two inverted repeat regions (IRs) of 26,132 bp. The cpDNA of *D. asperoides* contains 133 genes, including 89 protein-coding genes (PCG), 36 transfer RNA genes (tRNAs), and eight ribosomal RNA genes (rRNAs) The complete chloroplast genome of *D. asperoides* contains 133 genes (112 unique genes), among which 24 genes have one intron and four genes have two introns. Here, 20 genes were found duplicated in the every IR regions, including nine PCG species (*rpl22, rps19, rpl2, rpl23, ycf2, ndhB, rps7, rps12*, and *ycf1*), seven tRNA species (*trnI-CAU, trnL-CAA, trnV-GAC, trnI-GAU, trnA-UGC, trnR-ACG,* and *trnN-GUU*), and four rRNA species (*rrn16, rrn23, rrn4.5*, and *rrn5*). The overall nucleotide content of the chloroplast genome is A (Adenine) of 30.2%, T (Thymine) of 31.0%, C (Cytosine) of 19.7%, and G (Guanine) of 19.1%, with a total AT content of 61.2% and GC content of 38.8%.

Maximum-likelihood (ML) method analysis phylogenetic relationship with *D. asperoides*, the phylogenetic tree was constructed with MEGA X (Kumar et al. 2018) based on the amino acid sequences of 89 PCGs from 14 species chloroplast genome from GenBank to assess. ML analysis was performed using the MEGA X (Kumar et al. 2018) with 2000 bootstrap values replicate at each node based on GTR model. All of the nodes were inferred with strong support using the ML methods. The final ML tree was edited using the iTOL 4.0 online web (https://itol.embl.de/) (Letunic and Bork 2016). The ML tree (Figure 1) analysis result showed that the chloroplast genome of *D. asperoides* is closely related to *Dipsacus asper* (GenBank No. NC_039748.1 in the phylogenetic relationship. However, the cp genome of *D. asperoides* is very important to study this herb species and also can use for medicinal herb value research and clinical drug development in further.

**Figure 1.**
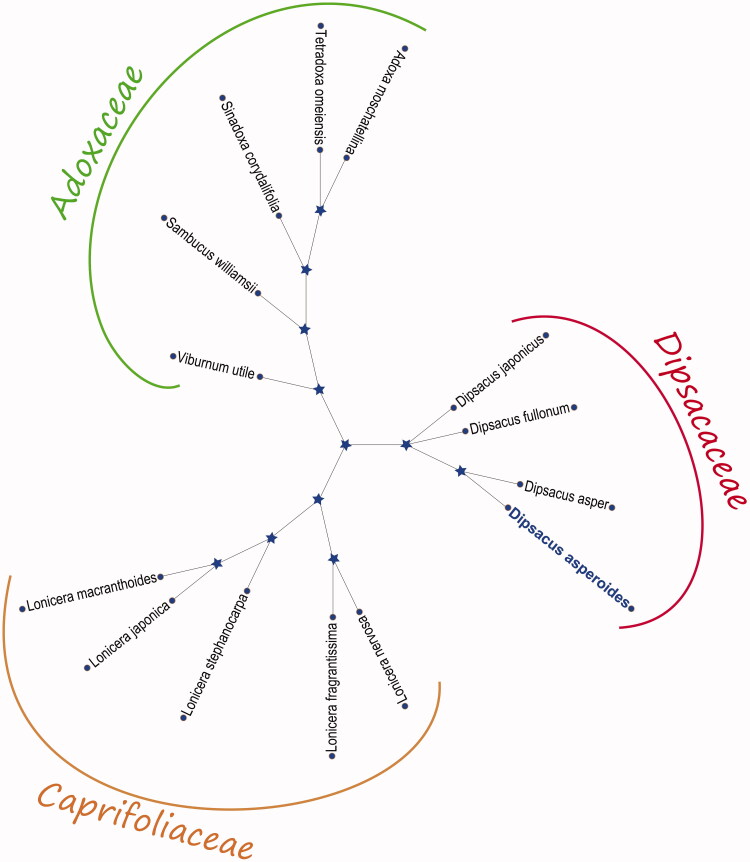
The phylogenetic tree was constructed using the Maximum-likelihood (ML) method and based on the amino acid sequences of 89 PCGs from 14 species chloroplast genome. The bootstrap values were based on 2000 replicates. GenBank accession numbers: *Adoxa moschatellina, NC_034792.1, Dipsacus asper NC_039748.1, Dipsacus fullonum MF350257.1, Dipsacus japonicus NC_039668.1, Lonicera fragrantissima MG738669.1, Lonicera japonica NC_026839.1, Lonicera macranthoides NC_040959.1, Lonicera nervosa MK176510.1, Lonicera stephanocarpa NC_037954.1, Sambucus williamsii, NC_033878.1, Sinadoxa corydalifolia, NC_032040.1, Tetradoxa omeiensis, NC_034793.1,* and *Viburnum utile, NC_032296.1.*
